# Very preterm neonates receiving “aggressive” nutrition and early nCPAP had similar long-term respiratory outcomes as term neonates

**DOI:** 10.1038/s41390-019-0514-5

**Published:** 2019-08-07

**Authors:** Polytimi Panagiotounakou, Rozeta Sokou, Eleni Gounari, Aikaterini Konstantinidi, George Antonogeorgos, Ioanna N. Grivea, Zoi Daniil, Konstantinos I. Gourgouliannis, Antonios Gounaris

**Affiliations:** 1 NICU General Hospital “Agios Panteleimon”, Piraeus, Greece; 2 Royal Alexandra Children’s Hospital, Brighton, UK; 3 NICU, University Hospital of Larissa, Larissa, Greece; 4 Respiratory Medicine Department, University of Thessaly School of Medicine, University Hospital of Larissa, Larissa, Greece

## Abstract

**Backround:**

The impact of the consistent implementation of “aggressive” nutrition by means of intensive early neonatal nutritional support up to 40–44 weeks postmenstrual age and the use of nasal continuous positive airway pressure (nCPAP) within the first hour of life on the respiratory function of very premature neonates (VPN) at school age is unclear.

**Method:**

Respiratory function was evaluated in 108 VPN and 70 term controls. Growth, frequency of lower respiratory tract infections, re-hospitalization, and spirometry were recorded up to 8–10 years of age. Comparison was carried out between the two study groups.

**Results:**

There was no significant difference in forced expiratory volume in 1 s and forced vital capacity at 8 years of age, and also in lower respiratory tract infections and re-hospitalization due to them, up to 8 years of age between preterm and term neonates. No significant difference was found in spirometry measurements neither between premature neonates with and without BPD nor between the two subgroups of preterms and term neonates.

**Conclusion:**

“Aggressive” nutrition, persistent nCPAP use, and their impact on early postnatal growth probably positively affect the respiratory function of our study population. These very encouraging results need to be confirmed by larger studies.

## Introduction

Respiratory system immaturity is the main cause of morbidity and mortality in very preterm infants, while bronchopulmonary dysplasia (BPD) represents a significant cause of long-term morbidity. In the “pre-surfactant era,” very preterm neonates with BPD would demonstrate increased airway obstruction compared to healthy term controls at school age.^1^ In the “post-surfactant era,” the survival rates improved significantly even for extremely premature neonates and led to high incidence of BPD^2^ and long-term respiratory complications during school age.^3^ Meta-analysis on very preterm neonates with and without BPD, born between 2000 and 2003, showed pulmonary function to be reduced when evaluated at 8–10 years compared to term controls.^4,5^ Feeding policies of very low birth weight (VLBW) neonates hospitalized up until early 2000s resulted in significant postnatal growth retardation. Lemons, et al.^6^ reported that 97% of VLBW neonates born in 1995–1996 were below 10th percentile at 36 weeks postmenstrual age (PMA). From the early 2000s, two policy changes have determined a new era in neonatology. In 2002, Ziegler et al.^7^ reported that “aggressive” nutrition, with early increased quantity of protein and calories, resulted in better growth. At the same time, early use of water-seal nasal continuous positive airway pressure (nCPAP)^8^ even in extremely low birth weight (ELBW) neonates resulted in decreased frequency and duration of invasive ventilatory support, reducing incidence of BPD and increasing weight gain.^9^ In the mid-2000s, Ehrenkranz et al.^10^ established that growth velocity, as expressed by weight and head circumference (HC), significantly affects psychomotor development. These are observations that support the hypothesis that nutritional interventions early in life probably decrease the risk of pulmonary complications (BPD) of very preterm neonates.^11^

Taking into account all the above data, we hypothesized that “aggressive” nutrition by means of intensive early neonatal nutritional support during neonatal period as well as early and persistent nCPAP in very preterm neonates would probably affect their short- and long-term respiratory outcome. The primary aim of the study was to evaluate respiratory function of very preterm neonates at school age by performing spirometry and to compare these measurements with term controls. As a secondary aim, it was decided to assess the growth and long-term pulmonary outcome as expressed with the frequency of lower respiratory tract infections and hospital readmissions up to 8–10 years of age. Furthermore, the impact of the presence of BPD on lung function in school age was investigated.

## Methods

### Subjects

**Fig. 1:**
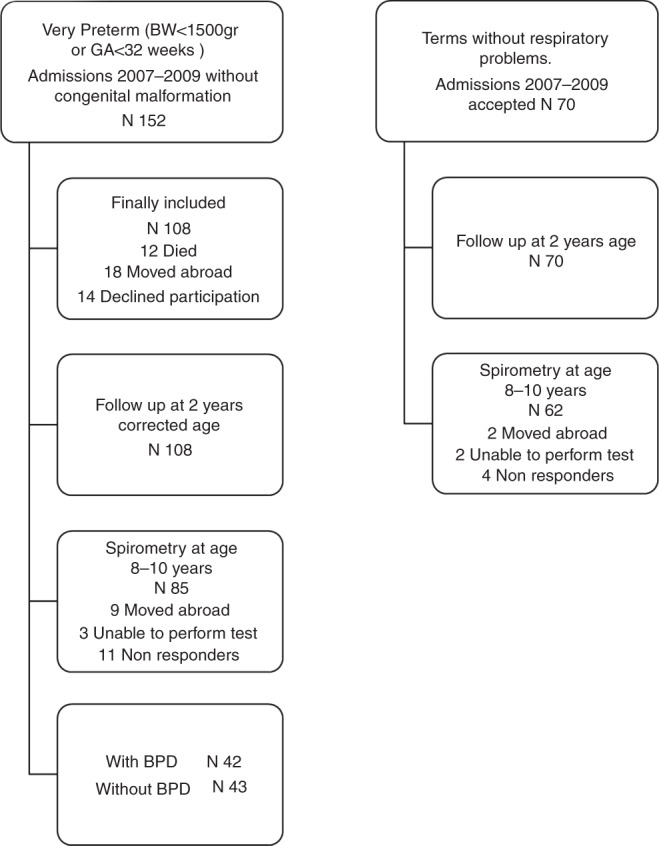
Flow chart of the study population

A short not validated questionnaire was developed, and in 2012 after approval of the hospital Ethical Committee and obtaining parental consent, the data for the study were collected. Data from the admission file and the outpatient long-term follow-up files of all study neonates admitted to our Neonatal Intensive Care Unit (NICU) between 2007 and 2009 were retrospectively collected. After 2012, data regarding the respiratory course of study neonates in the first 8–10 years of life were collected prospectively with the use of the questionnaire, clinical examination, and spirometry. The initial study population consisted of very preterm neonates with birth weight (BW) <1500 g and gestational age (GA) <32 weeks, without congenital abnormalities, admitted to our NICU during the study period (group A) and term neonates hospitalized in level 1 NICU for maximum 4 days in the same period (group B). Effort was made to avoid selection bias by including a sample of term neonates with minor medical problems, such as feeding difficulties during the 2 first days of life or neonatal jaundice, and excluding participants with major clinical entities, considered as confounding factors. Term neonates with congenital anomalies, respiratory problems, suspected or confirmed sepsis, and also small for gestational age were excluded. Data from 108 very preterm neonates and 70 term neonates were collected. Very premature neonates were divided into two subgroups: preterms with BPD (P-BPD) and preterms without BPD (P-non-BPD). As shown in the flow chart (Fig. 1), the families of 29 neonates were economical immigrants who returned to their countries (mainly Albania) in the aftermath of the Greek financial crisis and therefore lost to follow-up and were unable to participate in the study.

### Protocol

The Regional General Hospital “Agios Panteleimon” NICU—which is the regional perinatal center for neonates born in the West of Attica and the Aegean islands—has consistently implemented since 2006 the policies of “aggressive” nutrition and early and persistent use of water-seal nCPAP with permissive hypercapnia. Our interventions were aimed at trying to avoid significant postnatal growth retardation or, in the case of poor growth, at achieving the best optimal catch-up growth within 40–44 weeks PMA. The policies implemented are detailed below.

Every very preterm neonate received from birth total parenteral nutrition (TPN) with initial protein content of 2.5 g/kg/day and final protein content of 3.5–4.5 g/kg/day according to the GA in the first 5 days of life. Fat was added from the second day of life with final amount of 3 g/kg/day. For the uninterrupted administration of TPN, a central line catheter was inserted on day 1 of life, either umbilical venous catheter or peripherally inserted central catheter. In very premature neonates, milk feeds were introduced from the first or second day of life, mainly maternal milk or, if not available, preterm formula (Nestle Stage 1 with protein content 2.9 g/100 ml) as trophic feeds for 1–3 days, then increasing the amount with a rate of 24–36 ml/kg/day until achieving full enteral feeding at 200 ml/kg/day. Breast milk fortifier (Nutricia fortifier, protein 1.1 g/100 ml of milk) was added at 100 ml/kg/day of enteral feeds. If the growth rate achieved was unsatisfactory, especially of the HC, milk feeds were gradually increased up to 220–240 ml/kg/day. Very preterm neonates continued to receive fortified breast milk or post-discharge preterm formula after their discharge from NICU up until 44 weeks PMA or until HC reaches the 25th percentile.

Our respiratory policy was based on the introduction of early nCPAP.^9^ Very preterm neonates were started on water-seal nCPAP on admission to NICU within the first hour of life or the ones requiring intubation right after extubation. Very preterm neonates transferred from peripheral hospitals were started on nCPAP on admission to NICU. In 19 cases, there was a delay of up to 7 h as these neonates were transferred from islands of the Aegean Sea. Neonates remained on bubble nCPAP for the whole duration of their respiratory symptoms, while applying permissive hypercapnia (PaCO_2_ up to 70 mm Hg, as long as pH >7.22). Weaning attempts started after 2 weeks. Neonates developing tachypnea (respiratory rate >50/min), apneas, or FiO_2_ requirement >0.25 after discontinuation were placed back on nCPAP and further weaning was attempted after 48–72 h. Neonates remained on bubble nCPAP up until 33–34 weeks PMA (weight 1300–1400 g) or until they were in air or FiO_2_ <0.25. The prolonged use of nCPAP facilitated stable nutrition. BPD was defined as the need for O_2_ after 28 days of life and its severity was defined as mild, moderate, or severe according to the needs for O_2_ or respiratory support at 36 weeks PMA.^12^ Neonates with BPD and very preterm neonates <29 weeks GA received full course of palivizumab (Synagis).

### Measurements

Data regarding the duration of nCPAP after seventh day of life, duration of O_2_ administration, diagnosis, and severity of BPD were collected. The BW and HC of every preterm neonate of the study were retrospectively plotted on the Fenton Preterm Growth Charts^13^ and the mean growth of HC and body weight from birth to discharge (HC in cm/week and weight in g/day) were recorded. The very preterm neonates with BW and HC <10th percentile at birth and at discharge were noted. For all preterm neonates, weight, height, and HC at corrected age of 2 years and at the age of 8 years were recorded. Data regarding lower respiratory tract infections (need for antibiotics course or use of inhaled medication >3 days) and hospital admissions due to respiratory causes at 3 intervals were obtained. Initially and up to 2 years of age, data from their routine 3 monthly outpatient clinic follow-up appointments were collected. The second interval was between 2 and 5 years of age and the third between 6 and 8–10 years of age when spirometry was performed. During these periods, a dedicated member of the team was in regular contact with the parents and collected data via a questionnaire and from the mandatory pediatrician entries in the child’s health care record. For term controls, the same data for growth and respiratory infections and readmissions at the same intervals were documented.

The Spirolab spirometer of Mir technology was used for the spirometry. Ninety percent of all the measurements were performed at the neonatal outpatient clinic of the “Agios Panteleimon” Hospital according to the American Thoracic Society and the European Respiratory Society guidelines^14^ by the same technician who is an official technician of the Hellenic Respiratory Society and performs >2000 measurements/year. Respiratory function was expressed as the percentage of predicted values for height, weight, and sex. Flow–volume curves were obtained. Only forced vital capacity (FVC) and forced expiratory volume in 1 s (FEV1) in each neonate of the two groups were recorded. Children with spirometry values <70% predicted repeated the measurements in 3–6 months, assuming an issue with the child’s cooperation with the procedure.

### Data analysis

Prior to the implementation of the study design, sample size calculation was performed. An estimated sample size of at least 64 participants in each of the two study groups (preterm vs term neonates) was necessary in order to detect standardized differences between the means of the two groups of 0.5 *z*-score points, with statistical power 80%, using a significance level *a* = 0.05. All the continuous variables are presented as mean and standard deviation (SD), if normally distributed, or as median and range, otherwise. Categorical variables are presented as absolute and relative frequencies. Associations between continuous and categorical variables were assessed with *t* test (for mean comparison between two groups, aka full-term infants with all-born infants and preterm infants with and without BPD) and analysis of variance (for three group mean comparison), if the assumptions were met, or with Wilcoxon signed-rank test, if not. Chi-Square test, or Fisher’s exact test, where applicable, was used in order to assess possible relations between preterm vs term infants or in preterm infants between infants with BPD or not (non-BPD) with several anthropometric (weight, height, HC) or medical history characteristics (number of respiratory infections and hospital admissions) from birth up to 2 years, from 2 to 5 years, and from 6 to 8 years. All reported probability values (*P* values) were based on two-sided tests and compared to a statistically significant level of 5%. STATA 15 software was used for all the calculations (STATA Corp., College Station, TX).

## Results

Table 1 shows the characteristics of the neonates participating in the study. Very premature neonates with BPD had significantly lower BW and HC (*P* < 0.001, *P* < 0.001) and developed more severe complications such as patent ductus arteriosus, sepsis, necrotizing enterocolitis stage IIB—surgical, retinopathy of prematurity requiring laser therapy, and intraventricular hemorrhage grade >II during their NICU hospitalization compared to the rest of the premature neonates.

**Table 1:** Clinical characteristics of the neonates

	Term	Preterm	Preterm with BPD (P-BPD)	Preterm without BPD (P-non-BPD)	*P* value (P-BPD vs P-non-BPD)
GA (months), mean (SD) range			27.6 (1.7) 23^+5^–32	30.9 (1.2) 29–32	<0.001
BW (g), mean (SD)			1003.8 (186.2)	1346.6 (284.2)	<0.001
Head circumference (cm), mean (SD)			25.2 (1.7)	27.9 (1.7)	<0.001
Gender, male (%)	39 (62.9)	63 (58.3)	29 (59.2)	34 (57.6)	
Birth weight <10th percentile, *N* (%)	–	8 (7.4)	0 (0.0)	8 (13.6)	0.007
Head circumference at birth <10th percentile, *N* (%)		9 (8.3)	1 (2)	8 (13.6)	0.039
Head circumference <10th percentile at discharge, *N* (%)		5 (4.6)	2 (4.1)	3 (5.1)	0.805
Body Weight <10th percentile at discharge, *N* (%)		27 (25)	9 (18.4)	18 (30.5)	0.109
Antenatal corticosteroids, *N* (%)	–	55 (50.9)	24 (49)	31 (52.5)	0.748
RDS, *N* (%)		81 (75)	49 (100)	32 (54.2)	<0.001
BPD, *N* (%)	–	49 (45.3)	49 (100)	–	
Mild		17 (15.7)	17 (34.7)	–	
Moderate		12 (11.1)	12 (24.5)		
Severe		20 (18.5)	20 (40.8)		
nCPAP (median/range) in days			28 (64)	2(17)	<0.001
Postnatal corticosteroids for extubation, *N* (%)	–	5 (4.6)	5 (10.2)	–	
PDA significant, *N* (%)	–	17 (15.7)	15 (30.6)	2 (3.4)	<0.001
Conservative treatment		12 (11.1)	10 (20.4)	2 (3.4)	
Ibuprofen treatment		4 (3.7)	4 (8.1)	–	
Surgical ligation		1 (0.9)	1 (2)	–	
NEC (stage IIB–surgical), *N* (%)	–	1 (0.9)	1 (2)	–	
IVH grade III–IV, *N* (%)	–	9 (8.3)	8 (16.3)	1 (1.7)	0.012
Sepsis, *N* (%)	–	36 (33.3)	25 (51)	11 (18.6)	0.004
ROP–laser, *N* (%)	–	33–8 (30.5–7.4)	28–8 (57.1–16.3)	5–0 (8.5–0)	<0.001

*BPD* bronchopulmonary dysplasia, *BW* birth weight, *GA* gestational age, *IVH* intraventricular hemorrhage, *nCPAP* nasal continuous positive airway pressure, *NEC* necrotizing enterocolitis, *PDA* patent ductus arteriosus, *RDS* respiratory distress syndrome, *ROP* retinopathy of prematurity

Preterm neonates with BPD received mechanical ventilation for more days than those without BPD [days 9.5 (13.9) vs 0.91 (1.46), *P* = 0.000] and their hospital stay was longer [days 76.5 (27.77) vs 40.37 (15.13), *P* = 0.000]. The PMA of preterm neonates with BPD at discharge from the NICU was 38.6 (3.98) weeks, while preterm neonates without BPD were discharged at 36.2 (2.34) weeks (*P* = 0.001).

At birth, 7.4% of very premature neonates of our study had BW <10th percentile and 8.3% had HC <10th percentile. At discharge from NICU, 25% of very preterms had body weight <10th percentile and only 4.6% had HC <10th percentile. There were no significant differences at discharge regarding the percentage of very preterm neonates <10th percentile for body weight and HC between the two subgroups of very premature neonates. Neonates with BPD increased their body weight (mean, SD) from birth up to discharge by 21.8 (3.8)g/day, whereas neonates without BPD increased their weight by 21.1 (5.7)g/day (*P* = 0.515) and the HC by 0.81 (0.18)cm/week and by 0.82 (0.29)cm/week, respectively (*P* = 0.727).

Twenty-two very preterm neonates received caffeine after third day of life, 19 newborns with BPD for mean (SD) 6.9 (10.1) days, (median 19, range 1–36 days) and 3 newborns without BPD for 1, 10, and 18 days. Five very preterm neonates received corticosteroid therapy to aid extubation. None of the neonates with BPD received any corticosteroids for the prevention or treatment of BPD.

Growth of very premature neonates compared to term infants was significantly delayed at the age of 2 and 8 years as shown in Table 2, except for HC at school age (*P* = 0.09). The growth of very premature neonates with BPD compared to term infants was also significantly delayed at the age of 2 and 8 years (Table 2). There was no significant difference in growth between premature neonates with or without BPD at discharge, at 2 and 8 years of age, despite the fact that premature neonates with BPD were smaller and experienced more complications during their hospitalization.

**Table 2:** Spirometry, growth, respiratory infections, and admissions between groups

	Term	Preterm	P-BPD	P-non-BPD	*P* value		
					Term vs preterm	P-BPD vs P-non-BPD	Term vs P-BPD
FEV1^a^	92.6 (12.38)	91.0 (10.1)	89.8 (9.2)	92.2 (10.9)	0.418	0.285	0.231
FVC^a^	88.1 (11.6)	88.1 (10.5)	87.8 (9.3)	88.5 (11.6)	0.962	0.759	0.902
Head circumference (age 2 years)^a^	48.8 (1.5)	47.9 (1.7)	47.7 (1.3)	48.0 (2.0)	0.009	0.375	0.002
Weight (age 2 years)^a^	12.8 (1.5)	11.7 (1.8)	11.7 (1.5)	11.7 (2.0)	0.002	0.913	0.002
Height (age 2 years)^a^	90.1 (5.5)	86.2 (5.2)	86.4 (4.8)	86.0 (5.6)	<0.001	0.727	0.003
Head circumference (age 8 years)^a^	52.8 (1.7)	52.3 (1.6)	52.0 (1.4)	52.6 (1.8)	0.09	0.138	0.025
Weight (age 8 years)^a^	32.3 (8.2)	28.9 (6.4)	28.9 (6.7)	28.9 (6.3)	0.006	0.941	0.032
Height (age 8 years)^a^	133.5 (8.0)	130.1 (6.9)	129.1 (6.7)	131.1 (7.0)	0.007	0.204	0.005
Respiratory infections (age 0–2 years)^b^	0.0 (0–10)	0.0 (0–8)	1.0 (0–8)	0.0 (0–7)	0.173	0.133	0.048
Hospital admissions (age 0–2 years)^b^	0.0 (0–2)	0.0 (0–4)	0.0 (0–4)	0.0 (0–2)	0.080	0.489	0.056
Respiratory infections (age 2–5 years)^b^	0.0 (0–10)	0.0 (0–6)	0.0 (0–5)	0.0 (0–6)	0.035	0.977	0.061
Hospital admissions (age 2–5 years)^b^	0.0 (0–1)	0.0 (0–5)	0.0 (0–1)	0.0 (0–5)	0.089	0.233	0.03
Respiratory infections (age 6–8 years)^b^	0.0 (0–5)	0.0 (0–5)	0.0 (0–5)	0.0 (0–3)	0.154	0.418	0.407
Hospital admissions (6–8 years)^b^	0.0 (0–0.0)	0.0 (0–1)	0.0 (0–1)	0.0 (0–1)	0.234	0.974	0.230

*BPD* bronchopulmonary dysplasia, *FEV1* forced expiratory volume in 1 s, *FVC* forced vital capacity

^a^Mean (SD)

^b^Median (range)

Table 2 shows that there were no significant differences between subgroups P-BPD, P-non-BPD and group B in lower respiratory tract infections and respiratory admissions at the 3 periods checked except for lower respiratory tract infections at the age of 2–5 years, while when P-BPD were compared to term neonates the only statistically significant difference recorded was with regard to hospital admissions at the age of 2–5 years (*P* = 0.03).

**Fig. 2:**
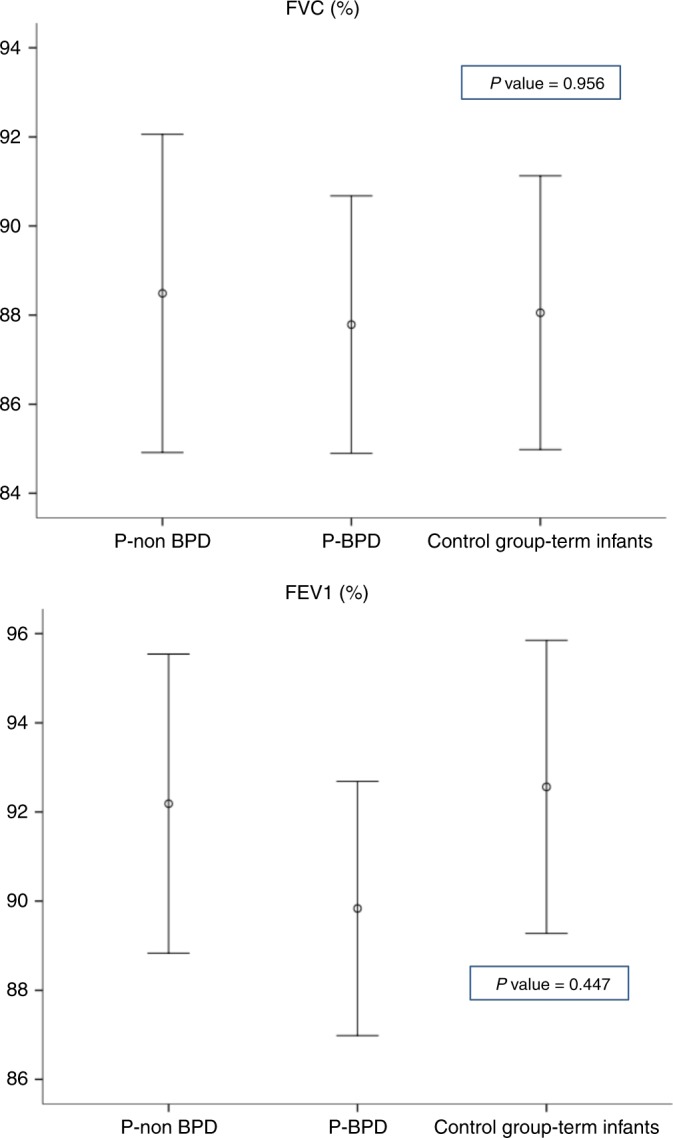
Comparison of forced vital capacity and forced expiratory volume in 1 s between the study groups

Statistical analysis of respiratory function at the mean age of 8.4 years (SD 0.8 years) for preterm and 8.3 years (SD 1.2 years) for term showed no significant difference in FEV1 and FVC between preterm and term neonates, preterm neonates with and without BPD, and the two subgroups of premature neonates and term neonates (Table 2, Fig. 2).

## Discussion

In this follow-up study, no statistical difference in pulmonary function was found regarding FEV1 and FVC of school age children who were born very preterm when compared with term controls. Lower respiratory tract infections and hospital admissions up to the age of 8 years were not significantly different between the above-mentioned groups.

Since it was first described, BPD has remained the main long-term respiratory complication in preterm neonates over the past 40 years. Efforts to prevent or reduce its incidence had no meaningful effect and BPD still affects 40% of very preterm neonates. Its characteristics have shifted and “new BPD” affects smaller neonates than before. Its incidence has virtually been unchanged in neonates <26 weeks gestation born in the UK in 1995 and 2006.^2^

Comparison of long-term respiratory outcomes of VLBW neonates with or without BPD in the pre- and post-surfactant era has yielded inconsistent results.^15^ There are studies showing considerable improvement in respiratory function in the post-surfactant era^16^ although that still remains considerably below that of term neonates. In another study, ELBW neonates born in 2005 had worse respiratory prognosis at 8 years of age compared to ELBW neonates born in 1991–92 and 1997.^17^

Improving survival rates from the beginning of the nineties resulted in maintaining or even increasing the incidence of BPD. At the same time, it was made clear that long-term respiratory problems are not only affecting preterm neonates with BPD but also VLBW and ELBW without BPD. In the post-surfactant era, respiratory evaluation at school age for very preterm neonates showed values significantly reduced compared to term controls, even for those without BPD.^3–5^ Halverson et al.^18^ in a study showed that very preterm neonates without BPD had differences from term controls, which were often comparable in severity with those of neonates with BPD.

Segerer et al.^19^ in a more recent study reaffirms the significant difference in respiratory function for VLBW and especially ELBW neonates not diagnosed with BPD and term controls. Further improvements in survival after the year 2000^20^ increased the numbers of VLBW neonates who survive without BPD and have expanded the pool of very premature neonates who need long-term respiratory monitoring.

The change in feeding modality after 2000 and the use of less invasive ventilation methods are the new norms in neonatology and effectively a new third era. Our results in this new era for the first time represent a positive message for the long-term prognosis of the respiratory system in VLBW neonates, even those with BPD. The multifactorial etiology of BPD makes it inherently difficult to precisely determine the factors that affect its natural history as some may still remain unknown. Despite that, the consistent use of “aggressive” nutrition, aiming at avoiding significant postnatal growth retardation or achieving catch-up growth within 40–44 weeks PMA, and the use of early bubble nCPAP, pioneered in early 2000 (Columbia Method),^8^ resulted in a better respiratory prognosis for all very preterm neonates admitted to the “Agios Panteleimon” NICU in the period 2007–2009 as evaluated at 8 years of age.

In our study, 25% of very premature neonates were <10th percentile for weight at discharge vs 7.6% at birth and 4.6% were <10th percentile for HC at discharge vs 8.3% at birth, with figures for weight at discharge improved compared to 97% in the NICH neonatal research network 1995–1996 data^6^ and to 44.05% at 36 weeks in a retrospective study of VLBW infants born during 2010–2014.^21^ The apparent close correlation of “aggressive” nutrition with neurodevelopment did not allow us to feed our very preterm neonates in a different way, so a group of preterms not subjected to “aggressive” nutrition could not be included in the study. Post-discharge growth was not significantly different between very premature neonates with or without BPD at 2 and 8 years, whereas it was significantly delayed compared to term infants, with the exception of HC at school age. Our results differ from those of Schmalisch et al.,^22^ who noted that the extent of somatic growth of very preterm infants with previous BPD lags behind those of preterm infants without BPD for the first 15 months of life.

Despite later delayed growth, the effect on the respiratory system, as determined by lower respiratory tract infections and hospital admissions up to the age of 8 years, was not significantly different both between the two subgroups of premature neonates and between them and the group of term neonates. These results differ significantly from those reported by Lamarche-Vadel et al.^23^ but are well in line with Pramana et al.,^24^ who showed no significant difference between the pulmonary outcomes (cough, wheezing, inhalation therapy, and re-hospitalization) of VLBW infants with and without BPD.

Our results were confirmed by the respiratory evaluation at 8 years of age and showed no significant difference in FEV1 and FVC between very premature and term neonates, very premature neonates with or without BPD, and between the two subgroups of premature and term neonates. These results can be interpreted as a combined effect of the “aggressive” nutrition and early nCPAP policies.

Since the nineties, Lucas et al.^25^ had noted significant improvement in the intelligence quotient (IQ) and neurodevelopment at the age of 7 years of very premature neonates receiving milk feeds with increased protein in up to 4 weeks after NICU discharge compared to neonates receiving the standard formula of that era. These findings led them to the theory that “early nutrition during critical windows in early life may have “programming” effects on long-term outcomes.”

Feeding policies up to 2000 resulted in significant postnatal growth retardation, as expressed by body weight, and HC during hospitalization was significantly correlated with adverse neurodevelopment outcomes in ELBW neonates.^10^

The situation changed in early 2000s after the introduction of increased protein and calorific intake through TPN^26^ and “aggressive” nutrition^7^ for the prevention of postnatal growth retardation. According to many studies, the positive impact of “aggressive” nutrition on growth up to discharge from NICU but especially between 26 and 34 weeks PMA results in significant improvement in IQ and neurodevelopment.^27,28^ Early aggressive parenteral and enteral nutritional support was associated with lower rates of death and short-term morbidities and improved growth and neurodevelopmental outcomes.^29^ That is likely to reflect improved alveolarization and respiratory function.

Nutrition influences prenatal lung development, directly affecting mechanisms of lung growth, but this also continues in postnatal life, especially in early infancy.

It has been long known through animal studies even after 2000 that intrauterine growth retardation showed to have long-term effect on the pulmonary structure. In neonates, intrauterine growth retardation was the sole factor implicated in the increased incidence of BPD in a study correlating intrauterine and perinatal factors with long-term respiratory prognosis.^30^ The postnatal undernutrition negatively affects lung maturation as this has been reported in rodent models, disrupting alveolarization and development of the bronchiolar epithelium.^31^ To what extent these experimental findings can be translated to humans is not known, but there is some evidence indicating that nutritional management of prematurely born infants may be involved in the pathogenesis of BPD and in greater long-term consequences on lung development as alveolarization continues until at least 2 years of age.^32^ Filbrun et al.^33^ concluded that “infants with BPD above average somatic growth showed significant greater improvement in respiratory tests (*P* < 0.05) than their peers.”

The early nCPAP implementation in our babies using the “Columbia method”^8^ and its duration in some preterms with unstable respiratory system until the 33–34 weeks PMA^34^ helped the uneventful continuation of enteral nutrition and possibly affect lung function through mechanisms already proven in animal models. The use of nCPAP for 2 weeks in an animal study has shown to increase the volume, weight, protein, and DNA content of the lungs comparing with controls (*P* < 0.01).^35^ More recently, daily CPAP use in newborn mice exposed to hyperoxia resulted in better respiratory mechanics (resistance, compliance), increased alveolarization, and reduced macrophage infiltration compared to the ones not on daily CPAP.^36^

In VLBW neonates, the early use of nCPAP for extended periods of time during respiratory instability and tolerance of higher PaCO_2_ levels during nCPAP resulted in decreased incidence of BPD, and in addition, these neonates had improved growth compared to controls.^9^ These results confirmed a recent meta-analysis as well as some previous ones that show a small but significant effect of nCPAP in the reduction of the incidence of BPD.^37^ nCPAP is known to improve gastric emptying in VLBW neonates and this mechanism potentially promotes feeding stability and growth of very premature neonates who remain on nCPAP.^38^

Permissive hypercapnia, the policy used during the nCPAP support in our study, is well documented as safe. It is considered appropriate for the weaning of intubated very preterm neonates from the ventilator.^39^ Also permissive hypercapnia does not increase the incidence of early complications and does not influence neurodevelopment at 18–22 months of corrected age of intubated neonates GA <29 weeks even in levels of PaCO_2_ up to 75 mm Hg.^40^

Limitations of the present study are its observational and retrospective design, the limited number of participants, and data from one single NICU. Strength of the study: single-center data ensure the consistent use of the policies and data recorded regarding growth, respiratory infections, hospital admissions, and spirometry up to 8 years.

In conclusion, although it is difficult to precisely determine the factors that affect the long-term outcome of BPD, due to its multifactorial nature, it appears likely that the “aggressive nutrition” of very premature neonates, the persistent use of nCPAP, and their impact on the achievement of early postnatal growth up to 40–44 weeks corrected age played an important role in the results of this single-center study. To the best of our knowledge, this is the first time that such encouraging results are shown regarding the long-term respiratory prognosis of very premature neonates (with or without BPD), although further large-scale comparative studies are necessary to verify these findings.
